# Online Multifamily Systemic Therapy after First Psychotic Episode

**DOI:** 10.1192/j.eurpsy.2023.678

**Published:** 2023-07-19

**Authors:** M. M. Selakovic, V. Pomini, A. Zartaloudi, D. Galanis

**Affiliations:** ^1^Department of psychiatry, GH Sismanoglio; ^2^1st Department of Psychiatry, National and Kapodistrian University of Athens Medical School; ^3^Mental Health Nursing Department, Nursing University of West Attica; ^4^Social worker, EPAPSY, Athens, Greece

## Abstract

**Introduction:**

In Greece, the Athens Multifamily Group Therapy Project (A- MFGT) provides systemic multifamily therapy to young adults after the first psychotic episode, with a purpose to deliver an early intervention program.

**Objectives:**

Few evidence is available regarding the viability of multifamily systemic therapy in an online setting in purpose of widely developing in e-mental health care (Borcsa et al., 2021). The members of ten families who participated at two online multifamily systemic groups for young adults after the onset of psychosis provided their opinions regarding their experience of 10-month therapeutic program through online platform, with two - hour sessions every 15 days.

**Methods:**

The members of the group described in an online form what they found helpful and/or unhelpful/harmful at three separate times: in the middle of the therapy process, at the end of therapy and at 6 months follow up period. The data collected was analyzed with an inductive, “data – driven”, form of coding based on the methodology of thematic analysis (Braun & Clarke, 2006). Themes represent the prevalence of what the participants found important in relation to the perceived effectiveness of online A- MFGT.

**Results:**

The members highlighted the impact of the online group process on family communication, reflected on advantages and difficulties offered by the online setting, described their emotions and the effect of diagnosis and hospitalization at individual and family level, valued the problem solving and empathy techniques in the group and identified the obstacles they encountered in the group sessions.

**Conclusions:**

Psychosis can affect all aspects of a person’s life, and without support and appropriate care, it can place considerable weight on the patient’s relatives, as well as the community in general. Our suggestion is that MFGT can be a viable way to support the whole system facing psychosis, with the aim of preventing relapse and implementing quality of life of all the participants.

**Disclosure of Interest:**

None Declared

